# New anti-α-Glucosidase and Antioxidant Ingredients from Winery Byproducts: Contribution of Alkyl Gallates

**DOI:** 10.1021/acs.jafc.3c03759

**Published:** 2023-09-28

**Authors:** Raúl Domínguez-Perles, Cristina García-Viguera, Sonia Medina

**Affiliations:** Laboratorio de Fitoquímica y Alimentos Saludables (LabFAS), CEBAS-CSIC, Campus of the University of Murcia-25, Espinardo, Murcia 30100, Spain

**Keywords:** winery byproducts, phenolipids, alkyl gallates, functionality, LC-MS/MS

## Abstract

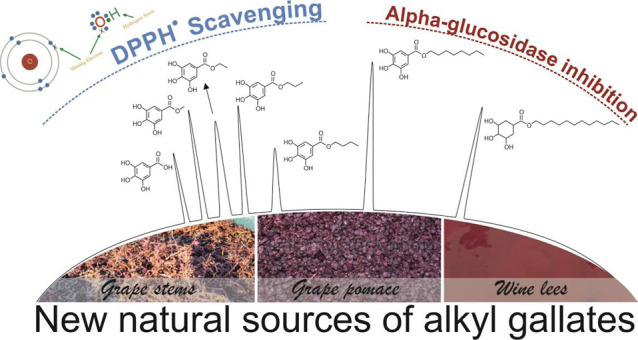

Wine-making activity entails the production of solid and semisolid byproducts (grape stems and pomace and wine lees) that negatively impact the environment and industrial sustainability. Their features as sources of bioactive compounds support valorization procedures for functional and healthy ingredients. This work uncovers the quantitative alkyl gallates (gallic acid esters, C1–C12) profile of fresh (freeze-dried) materials and the effect of oven-drying on their stability by UHPLC-ESI-QqQ-MS/MS. The functionality was established concerning DPPH^•^ scavenging and antihyperglycemic power. Wine lees exerted the highest high-free concentration of galloyl derivatives, ethyl gallate being the most abundant ester (3472.62 ng/g dw, on average). About the impact of the stabilization process, although as a general trend, the thermal treatment reduced the concentration, the reduction dimensions depended on the compound/matrix, remaining in valuable concentrations. Concerning radical scavenging, ze-dried stems and pomace displayed the highest capacity (24.11 and 18.46 mg TE/g dw, respectively), being correlated with propyl gallate (*r*^2^ = 0.690), butyl gallate (*r*^2^ = 0.686), and octyl gallate (*r*^2^ = 0.514). These two matrices exerted α-glucosidase inhibitory activity (1.58 and 1.46 units/L) equivalent to that of acarbose (a recognized α-glucosidase inhibitor). The newly described bioactive phytochemicals in winery residues (galloyl esters) and their correlation with functional traits allow for envisioning valorization alternatives.

## Introduction

Phenolipids or lipophenols are condensation products of fatty alcohols or fatty acids with phenolic compounds. These esterified phenolic compounds have received much attention due to their amphiphilic features with improved antioxidant ability relative to the parent compounds.^1–5^ Upon these chemical characteristics, lipophenols would display high solubility and stability in oily matrices and bioavailability, even surpassing the drawback enclosed to (poly)phenols for their application to functional products because of their polarity.^6,7^

Lipophenols based on gallic acid, especially alkyl gallates, have exhibited enhanced antioxidant, anti-inflammatory, antihyperglycemic, and antimicrobial properties relative to gallic acid.^4,8^ Beyond this, lipophenols have exhibited a parabolic antioxidant behavior (*aka* “cutoff” theory) characterized by an initial functional improvement, proportional to the alkyl chain length, until reaching a critical number of carbons, when the functionality drops.^9^ Accordingly, the length of the alkyl chain would define the biological scope of lipophenols, being the optimum alkyl chain length dependent on the food matrix and biological environment.^10,11^

Concerning the occurrence of lipophenols in plant materials, gallic acid and some galloyl esters, such as methyl gallate and ethyl gallate, have emerged as antioxidants present in diverse grape coproducts, including wine or vinegar, and also in winery byproducts.^12,13^ The residues enclosed in winemaking include solid and semisolid materials, namely grape pomace, stems, seeds, skins, or wine lees, are shortcomings for the sectorial industries that need to be addressed by designing proper valorization processes.^14^ Hence, the identification in these materials of new natural compounds with enhanced biological activities, such as alkyl gallates, could lead to valorization alternatives as food additives, nutraceuticals, functional ingredients, or cosmetics.^6^ Nonetheless, nowadays additional studies on the alkyl gallates’ profiles in oenological byproducts are still needed.

Based on these antecedents, this study aims at retrieving the quantitative alkyl gallate profile of fresh materials (grape stems and pomace, and wine lees) and the effect of a sustainable stabilization process (oven dehydration), compatible with industrial uses, on alkyl gallates. The present work also uncovers the contribution of these compounds to the antioxidant and antihyperglycemic power of winery byproducts. To establish the contribution of the alkyl gallates in association with their alkyl chain length, Spearman’s correlation was applied, and the actual relationship of methyl gallate, ethyl gallate, propyl gallate, butyl gallate, octyl gallate, and lauryl gallate with the biological activities recorded was described.

## Materials and Methods

### Chemicals and Reagents

The standards of gallic acid, alkyl gallic acid conjugates with different alkyl chain lengths (from C1 to C12) corresponding to methyl-, ethyl-, propyl-, butyl-, octyl-, and lauryl-gallates, and acarbose and the analytical kits (α-glucosidase activity) were purchased from Sigma-Aldrich (St. Louis, MO, USA). All LC-MS-grade solvents were purchased from J.T. Baker (Phillipsburg, NJ, USA). Deionized water was purified with a Milli-Q system (Millipore, Bedford, MA, USA).

### Plant Material and Stabilization Process

Stems and pomace from grape (*Vitis vinifera* L. var. “Monastrell”) and wine lees were provided by Via Elena winery (Jumilla, Murcia, Spain) in 2021. The referred winery byproducts were processed by freeze-drying (CHRIST vacuum concentrator 2–4D, Wolflabs, York, UK) for 120 h approximately (until constant weight) and oven-drying (applying the following descendent temperature gradient [time (min), temperature]: (0, 75 °C); (30, 72 °C); (60, 71 °C); (90, 69 °C); (120, 67 °C); (150, 65 °C); (180, 63 °C); (210, 61 °C); and (240, 61 °C), to complete the material’s stabilization, the residues were kept at 40 °C until constant weight. The period between sampling and processing was less than 4 h to avoid alteration of the phytochemical profile.

### Extraction of Gallic Acid Alkyl Esters

All freeze- and oven-dried materials (grape stems, grape pomace, and wine lees) were prepared following the procedure described by Milinčić et al.^12^ with slight modifications. Briefly, samples (100 mg) were first homogenized in 1 mL of ethanol, vortexed, and sonicated at 40 kHz for 1 h (Branson 3510MT sonicator, Sigma-Aldrich, St. Louis, MO) and maintained at 4 °C overnight. Afterward, the samples were centrifuged at 8750 *g*, for 5 min, at 4 °C, and filtered through a 0.22 μm PVDF filter (Millipore, MA, USA).

### Antioxidant Capacity Assay

The antioxidant activity was determined by the DPPH method, the most affordable alternative according to Baliyan et al.,^15^ adapted to a microscale.^16^ The antioxidant activity was determined by measuring the absorbance variation at 515 nm after 50 min of reaction in an Infinite M200 microplate reader (Tecan, Grödig, Austria). All reactions were developed by adding 2 μL of the hydroalcoholic extracts to wells containing 250 μL of the DPPH radical stock solution. The final volume of the assay was 252 μL. The results were expressed as millimolar Trolox Equivalents (mM TE).

### α-Glucosidase Inhibitory Activity

The α-glucosidase inhibitory activity was assessed using a Sigma-Aldrich commercial kit (Art. no. MAK123, St. Louis, MO, USA) according to the previously reported methodology.^17^ Briefly, samples (20 μL) were transferred to separate wells. Then, 200 μL of the master reaction mix [assay buffer pH 7.0/*p*-nitrophenyl-α-d-glucopyranoside substrate (25:1, *v*/*v*)] was added into each well. The initial absorbance was measured at 405 nm (A405_initial_) in an Infinite M200 microplate reader (Tecan, Grödig, Austria). Samples were incubated at room temperature for 20 min, and the final absorbance was monitored at the same wavelength (A405_final_). Acarbose was used at 1 mg/mL as a positive control, and the α-glucosidase activity was calculated as follows: “α-glucosidase activity (units/L) = [(A405)_final_ – (A405)_initial_/(A405)_calibrator_ – (A405)_water_] × 250 units/reaction volume (L)”.

### UHPLC-QqQ-MS/MS Analysis of Alkyl Gallates

Separation of gallic acid and alkyl gallates was performed using a UHPLC coupled with a 6460 triple quadrupole-MS/MS (Agilent Technologies, Waldbronn, Germany), using the analytical column BEH C18 1.7 μm (2.1 × 50 mm) (Waters, Milford, M.A.), according to the previously validated methodology.^18,19^ The column temperature was set up at 30 °C. The mobile phases consisted of deionized Milli-Q-water (LC–MS grade)/formic acid (99.9:0.1, *v*/*v*) (Solvent A) and acetonitrile (Solvent B). The injection volume and flow rate were 15 μL and 0.3 mL/min, correspondingly. The chromatographic separation of alkyl gallates was achieved upon the following linear gradient (time (min), % B): (0.0, 30%); (3.0, 98%); (6.0, 100%), and (6.1, 30%). The identification and quantification of the target analytes were achieved in the negative mode by multiple reaction monitoring (MRM) mass spectrometry, recording information quantification, and confirmation transitions for the corresponding analytes (Table S1). From the ethanolic stock solutions, successive dilutions at the μM level of concentration were prepared in methanol/deionized Milli-Q water (50:50, *v*/*v*) to facilitate ionization in the mass spectrometer. Data acquisition and processing were performed using Mass Hunter software, version B.08.00 (Agilent Technologies, Waldbronn, Germany). The identification of the target analytes was done resorting to the comparison of retention time (RT) (min), parent ions, and fragmentation patterns with authentic standards. Their quantification was completed by applying calibration curves freshly prepared each day with authentic standards.

### Statistical Analyses

Experiments were performed in triplicate (*n* = 3), and the concentrations presented as mean ± standard deviation (SD). According to the normal distribution pattern of the data (Shapiro–Wilk (<50 samples)), significant differences were set at *p* < 0.05 according to a one-way analysis of variance (ANOVA) and Tukey’s multiple-range test performed using the SPSS v. 28.0 software package (LEAD Technologies, Inc., Chicago, USA). The effect of the dehydration process (oven-vs freeze-dried) on the stability of the target analytes was established for each byproduct by a paired *t*-test. Existing correlations among concentrations and functionalities were analyzed by Spearman’s correlation tests.

A principal component analysis (PCA) was developed as a pattern recognition unsupervised classification method. The components score coefficient matrix, an output extracted by PCA and Varimax and Kaiser normalization as the rotation method, enabled us to show the weights of the variables studied. Significant correlations were at *p* < 0.05. The PCA was developed using Statgraphics Centurion XVI (StatPoint Technologies, Inc., 2010 USA).

## Results and Discussion

### Identification of Galloyl Alkyl Esters in Winery Byproducts

Gallic acid and galloyl esters are powerful antioxidants present in grape byproducts at high concentrations. Further knowledge concerning the profile of these functional compounds from winery byproducts opens up valorization opportunities that agrees with the circular economy policy of the European Commission for obtaining sustainable ingredients with enhanced biological scopes.^20^

When designing the analytical procedure for winery byproducts’ alkyl gallates, it was noticed that the characterization of such lipid esters could be dulled by the formation of artifacts during the extraction procedure, due to the interaction of ethanol or methanol with gallic acid.^6,21^ To avoid potential interferences, before analyzing the alkyl gallates content of winery byproducts, their formation during the extraction was determined to identify artifacts that could be falsely assumed as gallic acid derivatives naturally present in the plant material. To confirm this point, 5 mg of pure gallic acid were extracted with 1 mL of 6 different solvents (MeOH/deionized Milli-Q water (80:20, *v*/*v*); EtOH/deionized Milli-Q water (80:20, *v*/*v*); MeOH; EtOH; deionized Milli-Q water; and ethyl acetate) following matching conditions then applied with the byproducts’ samples (ultrasound-assisted extraction at 40 kHz, 25 °C for 1 h, maintained at 4 °C overnight, and centrifuged at 8750 *g*, for 5 min, at 4 °C). The analysis of the extracts obtained confirmed no formation of artifacts during the extraction process (data not shown) and, thereby, the consistency and robustness of the results retrieved on the alkyl gallates concentration in winery byproducts.

**Figure 1:**
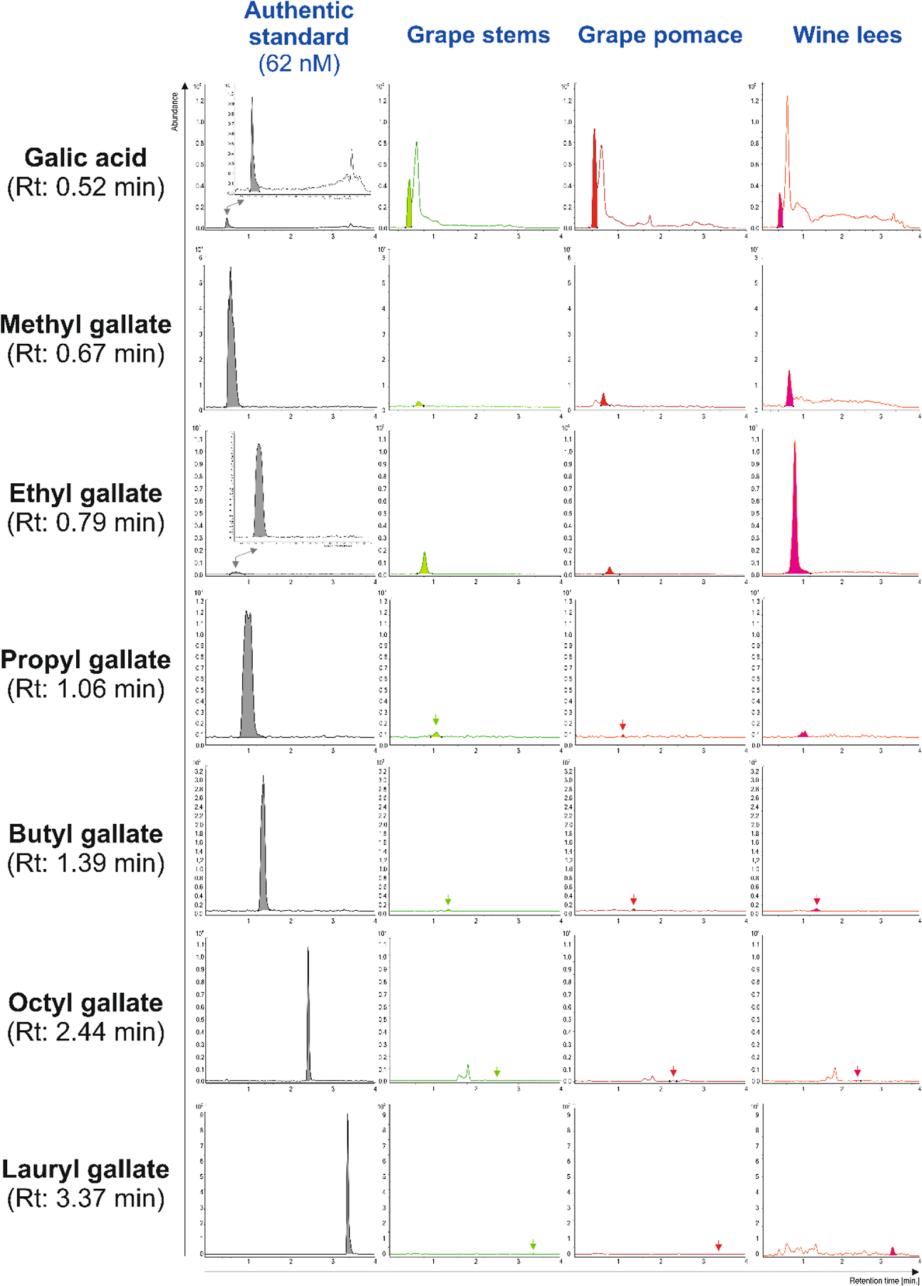
Representative UHPLC-QqQ-MS/MS chromatograms of the gallic acid and the alkyl gallates: methyl gallate, ethyl gallate, propyl gallate, butyl gallate, octyl gallate, and lauryl gallate provided authentic standards at 62 nM and scaled extracts of grape stems, grape pomace, and wine lees by with indication of their RT. CPS, charges per second.

Regarding the qualitative profile of gallic acid and its alkyl ester derivatives, the resolution and separation of both authentic standards and target compounds present in the winery residues under consideration were satisfactory and allowed monitoring of the target analytes according to the criteria mentioned before (Figure 1). The RT of alkyl gallates varied in parallel to the length of the alkyl-ester side-chain. Hence, longer elution times were observed for molecules with augmented lipophilicity in good agreement with previous descriptions for *p*-hydroxycinnamic acid derivatives.^22^ These results further demonstrated that the esterification of phenolic acids with fatty alcohols enhances their lipophilicity.^22^ Although the occurrence of gallic acid, methyl, and ethyl gallates have been previously reported in winery byproducts such as grape (*V. vinifera* L.) stems, seeds, and pomace,^13,23^ the present study enabled for the first time, as far as we know, the detection and identification of propyl gallate, butyl gallate, octyl gallate, and lauryl gallate in grape stems and pomace, and wine lees. From these newly reported alkyl gallates, to the best of our knowledge, only butyl gallate has been previously described as a natural product found in *Alchornea glandulosa*.^24^ Thereby, as far as we know, the present work is the first description of the natural occurrence of the complete range of alkyl gallates in plant materials (both edible products and byproducts), including butyl and lauryl gallates, which will allow to establish further comparison when additional data became reported.

### Quantitative Profile of Alkyl Gallates in Grape Stems, Grape Pomace, and Wine Lees

When analyzing the quantitative profile of galloyl lipophenols in freeze-dried byproducts, all the individual compounds were quantified in the three byproducts, except lauryl gallate, which remained in values below the limit of quantification (LOQ) in grape stems (Table 1). Moreover, freeze-dried wine lees exhibited the highest concentration of almost all galloyl derivatives except for gallic acid and octyl gallate that were found at the highest concentration in freeze-dried grape pomace and oven-dried grape stems, correspondingly (Table 1).

**Table 1:** Concentration (ng/g dw) of Gallic Acid and Its Esterified Derivatives in Freeze-Dried and Oven-Dried Winery By-Products (Grape Stems, Grape Pomace, and Wine Lees)^a^

		gallic acid esters						
winery byproducts	gallic acid	methyl gallate	ethyl gallate	propyl gallate	butyl gallate	octyl gallate	lauryl gallate	total alkyl gallates
Freeze-Dried Material								
grape stems	1750.00 ± 260.00 c^Z^	2.60 ± 0.14 c	750.29 ± 63.09 b	3.33 ± 0.40	10.91 ± 1.09 a	2.74 ± 0.63	<LOQ b	770.46 ± 63.03 b
grape pomace	4970.00 ± 240.00 a	17.57 ± 3.88 b	338.45 ± 11.58 c	1.76 ± 0.69	6.85 ± 0.65 b	1.20 ± 0.69	2.14 ± 1.15 b	367.96 ± 13.07 c
wine lees	2540.00 ± 160.00 b	27.91 ± 1.44 a	3852.99 ± 234.02 a	2.32 ± 0.87	11.40 ± 0.64 a	2.91 ± 1.09	13.11 ± 6.70 a	3910.65 ± 227.58 a
*p*-value	***^Y^	***	***	n.s.	***	n.s.	*	**
Oven-Dried Material								
grape stems	1620.00 ± 90.00 ab	2.76 ± 0.38 c	468.30 ± 15.31 b	1.30 ± 0.40	3.65 ± 0.17 b	1.39 ± 0.41 a	<LOQ b	477.35 ± 15.78 b
grape pomace	1470.00 ± 90.00 b	6.02 ± 0.59 b	144.41 ± 10.55 c	1.24 ± 0.91	2.78 ± 0.32 b	0.72 ± 0.09 b	0.21 ± 0.09 ab	155.10 ± 9.88 c
wine lees	1830.00 ± 80.00 a	21.14 ± 1.69 a	3092.24 ± 24.59 a	2.46 ± 0.47	8.45 ± 0.87 a	1.02 ± 0.18 ab	0.79 ± 0.41 a	3126.11 ± 26.44 a
*p*-value	**	***	***	n.s.	***	*	*	*
Comparison of Freeze-Dried and Oven-Dried Materials								
grape stems	n.s.	n.s.	***	**	***	*	n.s.	***
grape pomace	***	**	***	n.s.	***	n.s.	*	***
wine lees	**	**	**	n.s.	**	*	*	**

a ^Z^Data expressed as means ± SD (*n* = 3). Different lowercase letters indicate significant differences among winery byproducts for each separate treatment (oven-dried and freeze-dried) according to an ANOVA and the multiple range test of Tukey. The statistical differences between drying alternatives were examined by a paired Student’s *t*-test. ^Y^n.s., not significant; *, significant at *p* < 0.05; **, significant at *p* < 0.01; and ***, significant at *p* < 0.001.

Concerning the quantitative profile of galloyl derivatives in the separate freeze-dried byproducts, ethyl gallate was the phenolipid present at the highest concentration in wine lees (3852.99 ng/g dw, on average) that surpassed grape stems and pomace by 80.5 and 91.2%, respectively. The additional gallic acid lipophenols monitored (methyl gallate, lauryl gallate, butyl gallate, octyl gallate, and propyl gallate) were found at lower concentrations (2.32–27.91 ng/g dw) (Table 1). In addition, for freeze-dried materials, the concentration of these galloyl derivatives in wine lees was significantly higher than that recorded in grape stems for methyl gallate (a 90.6% lower) and lauryl gallate (<LOQ), while when comparing wine lees and grape pomace, significant differences were observed for methyl gallate, butyl gallate, and lauryl gallate (a 37.0%, 39.9%, and 76.0% lower, respectively). The comparison of grape stems and grape pomace evidenced significant differences for methyl and butyl gallates, more abundant in grape pomace and grape stems, correspondingly. The concentration of propyl gallate in freeze-dried residues was not significantly different. Finally, when monitoring the concentration of gallic acid (precursor), the highest concentration corresponded to grape pomace (4970.00 ng/g dw; Table 1). The calculation of the total burden of alkylgallates as the sum of the individual compounds found in concentrations higher than the LOQ informed on the following decreasing order for the winery byproducts under consideration wine lees (3518.38 ng/g dw, on average) > grape stems (623.91 ng/g dw, on average) > grape pomace (262.66 ng/g dw, on average) (Table 1).

The galloyl lipophenols’ profile in winery byproducts, with a preponderant relevance of wine lees, may be due to the fact that this residue is the result of the sugars’ fermentation by lactic acid bacteria (LAB) and yeasts. As a result of microbial metabolism, the phenolic profile undergoes critical modifications. This process has been documented in the scientific literature describing the formation of alkyl gallates, such as ethyl gallate, tentatively due to enzymatic reactions (e.g., lipase) of the ethanolic esterification of gallic acid by yeasts and LAB.^25^

Concerning the occurrence of galloyl lipophenols in plant-based foods and coproducts, and more specifically, ethyl gallate, and propyl gallate, higher concentrations have been reported in granadilla (*Passiflora ligularis*) and tamarind (*Tamarindus indica*) seeds of fermented flour using *Aspergillus niger*.^26,27^ More closely related to the matrices studied in the present work, other authors have observed valuable contents of ethyl gallate in grape seed, skin, stem, and whole pomace extracts (345.30, 86.30, 130.30, and 259.20 μg/g dw, respectively), because of the alcoholic fermentation, to which the grape pomace was subjected before analysis.^12^ These concentrations were above those recorded in the present study, possibly due to ethyl gallate being expressed as *p*-hydroxybenzoic acid equivalents and not being quantified with the appropriate standard. Beyond ethyl gallate, the methyl derivative has also previously been detected in grape byproducts (seed, stem, and pomace).^13^ However, to date, only one study performed by Mir-Cerdá et al. recovered ethyl gallate from winery lees at the average concentration of 2.10 μg/g dw,^28^ close to that found in this work (3.85 μg/g dw in freeze-dried samples) (Table 1). Nonetheless, to date, no comprehensive information on all alkyl gallates monitored in the present work has been described in the literature (e.g., regarding butyl and lauryl gallates) that limits significantly the scope of the discussion on this issue. From this point, the present study provides an update of the state-of-the-art by describing a more comprehensive quantitative profile of alkyl gallates from C1 to C12 in grape stems, grape pomace, and wine lees that could be a good and nontoxic alternative for synthetic food additives, such as synthetic propyl gallate, often used to prevent the free-radical peroxidation of lipids.^29^

### Effect of the Sustainable (Oven-Drying) Dehydration on the Alkyl Gallates

Valorizing winery byproducts as a source of compounds featured by an enhanced biological scope, such as alkyl gallates, has focused the attention of the scientific community aimed at exploiting them toward obtaining added-value coproduct. The lab-scale alternatives for processing plant materials may constitute uneconomical processes, not compatible with the industrial scale-up.^30^ Thus, more sustainable processes that overcome the constraints enclosed to freeze-drying as a stabilizing process are needed. Oven-drying constitutes a sustainable alternative that would allow the transference to the agro-food industry.^31^ However, before applying this alternative processing at the industrial level, the impact on the stability of the target compounds should be addressed.

From this premise, from the analysis of the galloyl ester derivative profile in winery byproducts stabilized by oven-drying, significant differences were identified relative to the freeze-dried materials (Table 1). While in the freeze-dried materials, lipophenols of gallic acid were found in the following average decreasing concentrations, ethyl gallate (1647.24 ng/g dw) > methyl gallate (16.03 ng/g dw) > butyl gallate (9.72 ng/g dw) lauryl gallate (5.08 ng/g dw) > propyl gallate (2.47 ng/g dw) > octyl gallate (2.28 ng/g dw), oven-drying dehydration modified this concentration order as follows, ethyl gallate (1234.98 ng/g dw) > methyl gallate (9.97 ng/g dw) > butyl gallate (4.96 ng/g dw) > propyl gallate (1.67 ng/g dw) > octyl gallate (1.04 ng/g dw) > lauryl gallate (0.33 ng/g dw) (Table 1). These differences were significant for ethyl gallate, propyl gallate, butyl gallate, and octyl gallate in grape stems; methyl gallate, ethyl gallate, butyl gallate, and lauryl gallate in grape pomace; and methyl gallate, ethyl gallate, butyl gallate, octyl gallate, and lauryl gallate in wine lees (Table 1).

These results evidenced that, although freeze-drying preserved gallic acid and galloyl esters derivatives to a higher extent than oven-drying, the materials dehydrated by the latter process also exhibited valuable concentrations (Table 1). In this regard, the concentrations of gallic acid, methyl gallate, and lauryl gallate in grape stems; propyl gallate and octyl gallate in grape pomace; and propyl gallate in wine lees were not significantly different (*p* > 0.05). These results are in good agreement with a previous study that reported enhanced preservation and extractability of bound phenolics from materials processed by freeze-drying compared to drying alternatives including oven-drying conducted by applying an optimized drying time (hours)/temperature conditions for grape pomace (27 and 10 h at 40 °C) and grape stems (17 and 5 h at 60 °C), microwave, and spiral flash dryer.^13^ Nevertheless, given that the application of freeze-drying at industrial levels is not a sustainable option in most cases, the reduction of the phytochemicals burden as a result of alternative dehydration processes should be assumed to a given extent marked by the maintenance of the functional traits. Moreover, freeze-drying is also enclosed to additional higher costs due to the packaging and storage requirements, due to increased porosity and hygroscopy, which could be associated with lower shelf life relative to conventional industrial processes such as that assayed in the present work.^32^

The modification of the abundance of individual alkyl gallates could be attributed to the *de novo* formation of gallic acid esters or their chemical transformations during the drying process. In this regard, as stated by ten Klooster et al. in a recent study on the thermal stability of gallic acid, this compound is hardly degraded when subjected to a temperature of 100 °C for 30 min^10^ All of this suggests that the gallic acid derivatives were not affected by the sample preparation. However, apart from temperature, other factors, including the abundance of enzymes (esterases/lipases) naturally present in winery byproducts, in close association with the enzymes’ selectivity and stability, the solubility of substrates, solvent polarity, and water content could affect the final concentration of alkyl gallates.^6^

According to these results, winery byproducts and especially wine lees could be suggested as valuable and sustainable sources of these compounds. According to the evidence on similar compounds in terms of structure–activity relationship,^33^ the esterifications featuring the chemical structure of galloyl esters could provide enhanced functionality compared to the native unesterified (gallic acid). Therefore, these results on establishing the quantitative profile of galloyl esters (C1–C12) of winery byproducts and the effect of dehydration for obtaining ingredients with high alkyl gallates’ concentration would allow for boosting the valorization of winery byproducts, including the less characterized one (wine lees).

### Antioxidant Activity of Winery Byproducts Rich in Alkyl Gallates

The assessment of winery byproducts stabilized (freeze- and oven-dried) allowed the establishment of different functional properties, namely DPPH^•^ scavenging (Table 2). In this regard, it is important to stress that the antioxidant determinations were done on extracts obtained by methods specific for alkyl gallates that minimize the interference with other potential antioxidant compounds present in winery byproducts (e.g., (poly)phenols), which enhances the significance of the correlations set up. Nonetheless, the extracts could also contain certain amounts of (poly)phenols, especially regarding those features by a low polarity (e.g., nonesterified flavonols). In this concern, according to previous comprehensive characterizations of the (poly)phenolic profile of the matrices under study developed by our own group,^23^ these compounds were only in significant concentration in wine lees where these could coadyuvate the antioxidant activity developed by alkyl gallates. Anyway, this fact should be overcome by analyzing in the frame of future investigations the actual concentration of (poly)phenols and alkyl gallates in final extracts addressed to be included as an ingredient in the development of new added-value commodities or even in the bioaccessible fraction of functional ingredients.

**Table 2:** DPPH^•^ Scavenging Capacity and α-glucosidase Inhibitory Activity of Freeze-Dried and Oven-Dried Winery By-Products (Grape Stems, Grape Pomace, and Wine Lees)^a^

winery byproduct	DPPH^•^ scavenging capacity (mg TE/g dw)	α-glucosidase inhibitory activity (units/L)
Freeze-Dried Material		
grape stems	24.11 ± 0.23 a^Z^	1.58 ± 0.04 b
grape pomace	18.46 ± 0.86 b	1.46 ± 0.29 b
wine lees	14.28 ± 0.73 c	123.50 ± 4.55 a
acarbose (positive control)		1.61 ± 0.10 b
p-value	***^Y^	***
Oven-Dried Material		
grape stems	11.45 ± 1.22 b	39.96 ± 1.39 b
grape pomace	10.64 ± 0.72 ab	4.65 ± 1.01 c
wine lees	13.95 ± 1.07 a	127.31 ± 0.25 a
acarbose (positive control)		1.61 ± 0.10 c
*p*-value	*	***
Comparison of Freeze-Dried and Oven-Dried Materials		
grape stems	***	***
grape pomace	***	**
wine lees	n.s.	n.s.

a ^Z^Data expressed as means ± SD (*n* = 3). Different lowercase letters indicate significant differences among winery byproducts for each separate treatment (oven-dried and freeze-dried) according to an ANOVA and the multiple range test of Tukey. The statistical differences between drying alternatives were examined by a paired Student’s *t*-test. ^Y^ n.s., not significant; *, significant at *p* < 0.05; **, significant at *p* < 0.01; and ***, significant at *p* < 0.001.

As expected on the basis of the galloyl esters’ burden of freeze- and oven-dried winery byproducts, the formers displayed the highest DPPH^•^ scavenging power (14.28–24.11 mg TE/g dw) in comparison with the oven-dried materials (11.45–13.95 mg TE/g dw) (Table 2), in good agreement with the relative concentration of alkyl gallates in matrices processed by freeze- and oven-drying (Table 1). However, these differences were only statistically significant for grape stems and pomace (both at *p* < 0.001).

Concerning freeze-dried materials, when comparing the different matrices, grape stems provided the significantly highest radical scavenging activity (24.11 mg TE/g dw), followed by grape pomace (18.46 mg TE/g dw) that, in turn, exhibited a stronger antioxidant activity than wine lees (40.8 and 22.8% lower than grape stems and grape pomace, correspondingly; Table 2). The high antioxidant activity of grape stems has been previously described resorting to in vitro models,^16^ and tentatively attributed, based on the compositional knowledge at the time, to procyanidins polymers, as well as flavanol monomers and oligomers. Nonetheless, recently, the contribution of other phenolic compounds as gallic acid and ethyl gallate (the latter recently described in grape stems) should not be ruled out.^34^ In the assessment of winery byproducts stabilized by procedures compatible with industrial scale-up (oven-dried), the highest antioxidant activity corresponded to wine lees that surpassed the capacity of grape stems and grape pomace by 20.8%, on average, while no significantly different DPPH^•^ scavenging capacity was found between grape stems and grape pomace (*p* > 0.05). This result may be due to the similar concentrations of gallic acid and most galloyl esters (methyl gallate, propyl gallate, butyl gallate, and octyl gallate) (Table 1). In this context and according to the gallic acid and galloyl esters burden of the diverse matrices assessed, alkyl gallates may provide a valuable contribution to the overall radical scavenging activity and the different evolution of the radical scavenging capacity of the three separate matrix after the dehydration process. In this regard, the phenolic profile of wine lees, formed to some extent with the concourse of the yeasts metabolism could be more resistant to the thermal treatment applied with stabilization purposes, as recently demonstrated by Costa-Pérez et al. (2023).^23^ Specifically, (poly)phenolic compounds featured by low polarity (e.g., unesterified quercetin or kaempferol), could effectively contribute to the higher radical scavenging power of this matrix. Beyond this, the interaction of the antioxidant compounds present in the three matrices under consideration with additional components of wine lees (present in lower concentration in grape stems and pomace) during the manufacturing process could give rise to the formation of new highly antioxidant compounds (such as alkyl gallates) that would entail enhanced antioxidant potential in comparison with grape stems and pomace. Thus, they could act as antioxidants in a variety of ways that include quenching reactive oxygen species, inhibiting various prooxidant enzymes involved in their production, as well as chelating divalent metal ions.^35^

Hence, alkyl gallates should be considered multifunctional antioxidants by molecular mechanisms directly or indirectly explained by their chemical structure and, specifically, by their alkyl side chain, the length of which is closely related to the antioxidant power.^35^ In this concern, recently, alkyl chain length has been demonstrated as a critical factor for the modulation of the antioxidant activity of gallic acid esters in spray-dried emulsions, in which octyl gallate seems to be most effective.^10^ This allows envisaging more successful strategies in the prevention of peroxidation reactions in lipid-based food products, where the efficiency of polar antioxidants is significantly lower.^3^ In this regard, some alkyl gallates, such as lauryl gallate, have demonstrated indirect preventive antioxidant properties, being capable of inhibiting mitochondrial lipid peroxidation that constitutes a valuable advantage relative to the phenolic precursor (gallic acid). The different biological scopes of both types of compounds reinforce that the hydrophobic alkyl chain is essential for that particular antioxidant property.^36^ Indeed, according to a recent report on the topic, the chemical structure of the gallate moiety has an excellent electron-donating capability directly related to the radical scavenging power.^8^

In this frame, winery byproducts as an economic and rich source of alkyl gallates would constitute sustainable ingredients with a valuable amphiphilic phytochemical content based on alkyl gallates, thus suitable to lower lipid oxidation in fat-based products, extending their shelf life and conferring their healthy properties. In addition, these natural matrices with high levels of antioxidant alkyl gallates could replace the synthetic antioxidant additive such as propyl gallate (E310) extensively used in the food containing fats to prevent oxidation approved by the European Food Safety Authority (EFSA) in its Scientific Opinion on the re-evaluation of propyl gallate as a food additive, at a maximum level ranging from 25 to 400 mg/kg.,^37^ as well as in cosmetics and pharmaceutical industries.^29^

### α-Glucosidase Inhibitory Activity of Winery Byproducts

Since the enzyme α-glucosidase is responsible for the hydrolysis of carbohydrates and the enhanced absorption of glucose through the intestine epithelium, its inhibition has been considered an effective way of decreasing postprandial hyperglycemia.^38^ Hence, in close connection with previous studies describing the α-glucosidase inhibition by (poly)phenols, alkaloids, or terpenoids,^39^ the assessment of alkyl gallates on their capacity to delay glucose absorption and, thus, provide antidiabetic advantages deserves to be explored. Based on this premise, the winery byproducts (grape stems, grape pomace, and wine lees) were evaluated on the α-glucosidase inhibitory activity (units/L) (Table 2). In this assay, acarbose, a pseudotetrasaccharide inhibitor of α-glucosidase extensively studied, was used as an antiglucosidase drug.^39^ When comparing the inhibitory effect of alkyl gallate extracts of winery byproducts with that developed by α-glucosidase, it was found that freeze-dried grape stems and grape pomace displayed the most powerful inhibition (1.58 and 1.46 units/L, correspondingly). This was comparable to that of this well-known α-glucosidase inhibitor, acarbose (1.61 units/L), with no significant statistical differences between these winery residues and acarbose. On the other hand, wine lees presented an almost lack of hypoglycemic activity (Table 2). Interestingly, after oven-drying, grape stems and grape pomace reduced significantly their α-glucosidase inhibitory activity, being identified significant differences attributable to the dehydration process (*p* < 0.001 and *p* < 0.01, respectively). The results retrieved in the present work concerning the α-glucosidase inhibition were consistent with previous those reported in recent studies that identified grape pomace as one of the winery byproducts with strong inhibitory activity on mammalian intestinal α-glucosidases.^40,41^

To the present date, the hypoglycemic activity resulting from the α-glucosidase by winery byproducts has been associated with their (poly)phenolic content, and specifically with the phenolic acids and alkyl gallates, which are powerful inhibitors of this enzyme thanks to their hydroxyl groups, crucial in the inhibition effect.^42,43^ In this work, the characterization of a broader range of galloyl esters has revealed promising α-glucosidase inhibitory activity of these compounds, in good agreement with a previous report that demonstrated the strong inhibitory activity against α-glucosidase of gallic acid both in vitro and in vivo assays, with IC50 value (24.3 mol/L), even higher than the attributed to acarbose (59.5 mol/L).^44^ Likewise, the hypoglycemic effect of extracts obtained from “Merlot” grape pomace resorting to the inhibition of α-glucosidase has been demonstrated upon in vitro, in silico, and in vivo, as well as the capacity to prevent high fat diet-induced type 2 diabetes.^45,46^

In addition to the hypoglycemic effect of the gallic acid and its alkyl esters of different winery byproducts, other lipophenols isolated from additional vegetal matrices, such as stem and bark extracts of *Terminalia superba* (Combretaceae), revealed significant α-glucosidase inhibition activity of gallic acid and methyl gallate.^47^ In the same way, methyl gallate isolated from *Bouea macrophylla*, commonly known as marian plum or plum mango, exhibited potent inhibition of α-glucosidase with an IC50 value of 71.0 μM, further demonstrating that this compound might account for the inhibitory activity.^48^ Moreover, propyl gallate of green tea extracts (IC50 = 11.5 mol/L) exhibited strong α-glucosidase inhibition. Altogether, these findings would reveal that the presence of the gallate group in the chemical structure is key for an efficient inhibition of α-glucosidase.^49^ This activity could be due to the enzymatic inhibition developed by gallic acid and galloyl derivatives by competing directly with the substrate for the active binding site of the enzyme.^50^

The hypoglycemic effect, could be also due to the inhibition of starch digestion by gallic acid and alkyl gallates as has previously been reported in the literature.^8^ This inhibitory ability decreased in a reverse form with the alkyl chain length, as demonstrated by assessing how medium alkyl chain gallates (butyl and octyl gallates) were more efficient in inhibiting the enzymatic digestion of starch. In this aspect, the lower capacity of longer alkyl chain gallates (hexadecyl (C16) and octadecyl (C18) gallates) has been attributed to their low solubility and to the susceptibility to self-association in solution.^8^ Thereby, the alkyl gallates with intermediate chain length (C4–C8) provide the optimal polarity to guarantee the water solubility required to interact with enzymes, providing a modulating the glycemic response.^10^

### Principal Components and Correlation Analyses

To set up the relationship existing between the levels of gallic acid and alkyl gallates and the radical scavenging and α-glucosidase inhibition of freeze- and dried grape stems, grape pomace, and wine lees, Spearman’s correlation was developed. As a result, positive correlations between DPPH^•^ scavenging and the concentration of propyl gallate (*r*^2^ = 0.690; *p* < 0.01), butyl gallate (*r*^2^ = 0.686; *p* < 0.01), and octyl gallate (*r*^2^ = 0.514; *p* < 0.05) were achieved. Thus, these results support the previous discussion on the relevance of the alkyl chain length for reaching the highest antioxidant activity, as medium-size alkyl gallates (C3–C8) were the best antioxidants compared to short-size alkyl gallates (C1–C2) and lauryl gallate (C12) in good agreement with previous descriptions in the literature.^33^ The valuable activity of such medium-size alkyl gallates (C4, butyl gallate) has been proposed as a result of the hydrophobicity that this feature confers to the alkyl gallate, which would be a key factor for the antioxidant efficiency.^19^ In this regard, propyl gallate has been reported as a very efficient peroxyl radical scavenger in both aqueous and lipid media, where trihydroxy substitutions in the propyl gallate molecule provide strong antioxidant traits, being the *para*-hydroxyl the most important group in comparison with the ester one.^51^ Concerning the antioxidant activity, it is also important to stress that gallic acid has exhibited a high synergy with ethyl and lauryl gallates, which has been also described between ethyl and lauryl gallates.^52^ This fact allows for envisaging synergies among compounds with different chemical properties or matrices (e.g., winery byproducts), rich in alkyl gallates.

**Figure 2:**
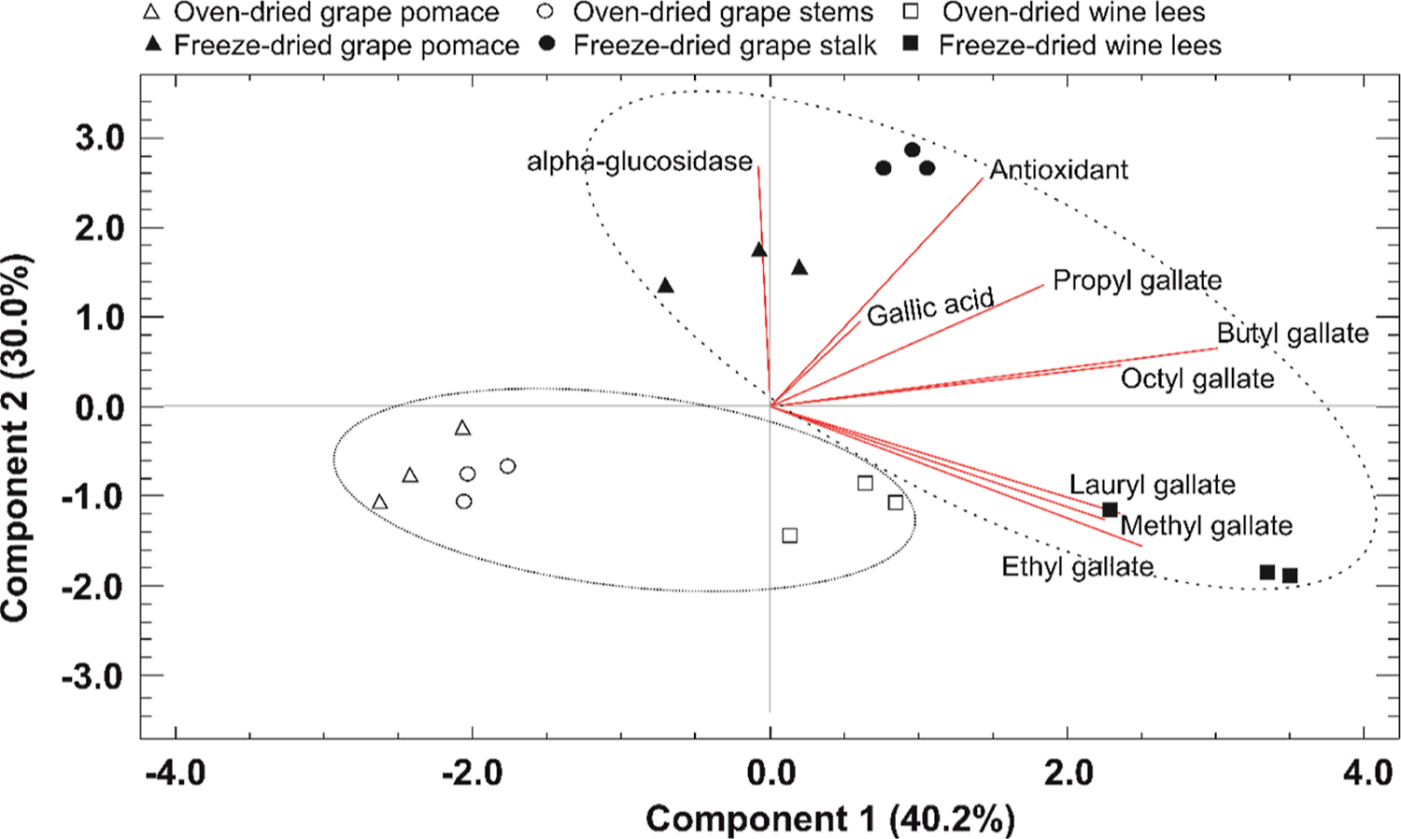
PCA of the concentration gallic acid and alkyl gallates in freeze-dried and oven-dried winery byproducts (grape stems, pomace, and wine lees), as well as, antioxidant activity and α-glucosidase inhibitory property. Plots of principal components of the first two components account for at least 70.2% of the total variance.

In addition, the PCA performed on the data matrix that included gallic acid, alkyl gallates, and biological properties (antioxidant activity and α-glucosidase inhibitory property) revealed acceptable scores for the two principal components (PC1 and PC2), providing 70.2% explained variance (Figure 2). Moreover, PCA allowed the reduction of the data dimension, showing the clustering into two main groups, colored (freeze-dried samples) and noncolored (oven-dried samples). The joint analysis of all results noticed that freeze-dried stems and pomace were the matrices with the highest biological scopes, findings in line with a previous report that informed the grape pomace extracts display significant antioxidant and hypoglycemic effects.^40,41^ Interestingly, these dual functionality could provide complementary and alternative roles in managing the oxidative stress associated with type 2 diabetes and other disorders.^53^

As concluding remarks, the present work outlined the importance of the quantitative alkyl gallate profile of oenology byproducts (grape stems, grape pomace, and wine lees) as a critical input to the management of winery residues toward new ingredients with differentiated functionality (antioxidant and antidiabetic). In this regard, both freeze- and oven-drying constitute feasible alternatives to obtain stabilized materials with a valuable content of galloyl esters, with the latter being the more sustainable choice. Our results displayed that freeze-dried stems and pomace were the matrices with the highest biological potential. Nonetheless, wine lees exhibited the highest concentration of specific alkyl gallates, namely, methyl and ethyl gallates, regardless of the dehydration process carried out. Based on these results, depending on the functionality intended, one type of winery byproduct or another one, as well as mixtures thereof, concerning the content of alkyl gallates, could be used to obtain stabilized ingredients and apply such as coadjutants in different physio-pathological processes. Despite the current knowledge gathered on the biological interest of alkyl gallates, continuing to investigate the mechanisms of action of these compounds is still needed, which is especially suggested by the data presented in this study. In this concern, our findings provide a new perspective that will boost the identification of new valorization alternatives for winery wastes, for instance, in the development of new healthy and sustainable foods with improved functionalities in the frame of metabolic disorders (e.g., type-2 diabetes, gestational diabetes, and other metabolic disorders). Nevertheless, nowadays, the gap of knowledge concerning the absorption, distribution, metabolism, and excretion of galloyl esters need to be addressed in order to develop novel functional foods based on winery byproducts in the frame of the circular economy.

## Supporting Information Available

MRM parameters, limit of detection, and LOQ of the separate gallic acid and alkyl gallates obtained by UHPLC-QqQ-MS/MS in negative ionization mode (PDF)
(PDF)
