# Supplementary material for: New anti-α-Glucosidase and Antioxidant Ingredients from Winery Byproducts: Contribution of Alkyl Gallates
Source: J Agric Food Chem. 2023 Sep 28;71(40):14615–25. doi: 10.1021/acs.jafc.3c03759 (PMC10571075; doi:10.1021/acs.jafc.3c03759)
Supplement: Supplementary file 1 — jf3c03759_si_001.pdf [file jf3c03759_si_001.pdf]

SUPPORTING INFORMATION

New anti-α-glucosidase and antioxidant ingredients from winery by-products: Contribution of alkyl gallates

Raúl Domínguez-Perles, Cristina García-Viguera*, Sonia Medina

Laboratorio de Fitoquímica y Alimentos saludables (LabFAS), CEBAS-CSIC, Campus of the University of Murcia-25, 30100 Espinardo, Murcia, Spain.

* Corresponding author.

E-mail address: cgviguera@cebas.csic.es (C. García-Viguera).

Table Supplementary 1. Multiple reaction monitoring parameters, limit of detection, and limit of quantification of the separate gallic acid and alkyl gallates obtained by UHPLC-QqQ-MS/MS in negative ionization mode.

**Compound**	**Retention time (min)**	**Molecular weight**	**Precursor ion (*m/z*)**	**Product ion (*m/z*)**	**Fragmentor (V)**	**Collision energy (eV)**	**LOD (ng/mL)**	**LOQ (ng/mL)**
Gallic acid	0.52	170.12	169	151; **125**; 79	90	9	0.663	1.327
Methyl gallate (C1:0)[a]	0.67	184.15	183	169; **124**;78	90	9	0.166	0.359
Ethyl gallate (C2:0)	0.79	198.17	197	169; 151; **124**	90	9	0.178	0.386
Propyl gallate (C3:0)	1.06	212.20	211	169; **124**; 111	90	9	0.191	0.414
Butyl gallate (C4:0)	1.39	226.23	225	169; **124**; 111	90	9	0.204	0.441
Octyl gallate (C8:0)	2.44	282.33	281	169; 140; **124**	90	9	0.254	0.551
Lauryl gallate (C12:0)	3.37	338.44	337	169; **124**; 111	90	9	1.320	2.640

[a] The information in parentheses is referred to the alkyl chain length linked to gallic acid. Product ion (*m/z*) in bold was used for quantification. LOD: limit of detection; LOQ: limit of quantification;
