# Supplementary material for: New anti-α-Glucosidase and Antioxidant Ingredients from Winery Byproducts: Contribution of Alkyl Gallates
Source: J Agric Food Chem. 2023 Sep 28;71(40):14615–25. doi: 10.1021/acs.jafc.3c03759 (PMC10571075; doi:10.1021/acs.jafc.3c03759)
Supplement: Supplementary file 2 — jf3c03759_si_002.pdf [file jf3c03759_si_002.pdf]

Journal of Agricultural and Food Chemistry

New anti-α-glucosidase and antioxidant ingredients from winery by-products: Contribution of alkyl gallates

HIGHLIGHTS

- Grape stems, grape pomace, and wine lees are sustainable sources of alkyl gallates
- Wine lees exhibited the highest content of methyl, ethyl, and lauryl gallates
- The dehydration process (freeze- *vs* oven-drying) provides different capacities to preserve alkyl gallates
- Freeze-dried grape stems and pomace displayed the best antioxidant and hypoglycemic activities
